# Cardioversion-Induced Takotsubo Cardiomyopathy

**DOI:** 10.7759/cureus.64349

**Published:** 2024-07-11

**Authors:** Ryan Clydesdale, Shivani Reddy, Jagadeesh K Kalavakunta, Jose Ricardo Po

**Affiliations:** 1 Internal Medicine, Western Michigan University Homer Stryker M.D. School of Medicine, Kalamazoo, USA; 2 Cardiology, Ascension Borgess Medical Center, Kalamazoo, USA

**Keywords:** atrial fibrillation, cardioversion, stress cardiomyopathy, takotsubo cardiomyopathy, cardiac imaging

## Abstract

Takotsubo cardiomyopathy, also known as stress-induced cardiomyopathy or "broken heart syndrome," is a rare and reversible condition characterized by transient left ventricular dysfunction. It is typically triggered by acute emotional or physical stressors. Here, we present a unique case of TCM occurring in a 77-year-old woman following cardioversion for persistent and symptomatic atrial fibrillation. The patient underwent uncomplicated cardioversion with recent imaging showing intact global systolic function. She presented four days post-procedure for chest pain, shortness of breath, and peripheral edema. A repeat echocardiogram showed a marked decrease in cardiac function evidenced by an ejection fraction of 20-25%. The patient was readmitted and managed with IV diuretics. Symptoms resolved within three days and the patient showed improved cardiac function on imaging prior to discharge.

## Introduction

Takotsubo cardiomyopathy (TCM), also known as stress-induced cardiomyopathy or "broken heart syndrome," is a reversible condition characterized by transient left ventricular dysfunction [1]. It mimics the clinical presentation of acute coronary syndrome but is characterized by the absence of significant coronary artery disease [1]. This condition typically presents with chest pain and dyspnea [2], electrocardiographic changes, and elevated cardiac biomarkers, resembling myocardial infarction, yet it is distinguished by its unique pathophysiological mechanisms [3]. It is typically triggered by acute emotional or physical stressors that induce catecholamine release [1]. Other risk factors include genetics and estrogen deficiency, with the most common patient population being postmenopausal women [4].

Atrial fibrillation (AF) is a common cardiac arrhythmia characterized by irregular atrial activity and rapid ventricular response, often requiring therapeutic interventions to restore normal sinus rhythm. One such intervention is electrical cardioversion, which involves the delivery of synchronized electrical shocks to the heart to terminate AF and restore the heart's normal rhythm. While cardioversion is considered a safe and effective procedure, it is not without potential risks and complications, including the induction of stress cardiomyopathy. However, stress-induced cardiomyopathy following cardioversion is extremely rare [5].

In this case report, we describe a case involving a 77-year-old female found to have a rare case of TCM following cardioversion for persistent AF.

## Case presentation

A 77-year-old obese Caucasian woman with a past medical history of hypertension, hyperlipidemia, and nonvalvular AF presented initially with complaints of sporadic heart palpitations. The palpitations worsened and later on, became associated with fatigue and a decrease in exercise capacity. Her baseline is NYHA (New York Heart Association) class shortness of breath but no angina. Holter monitoring for 48 hours revealed a 100% burden of AF with an average heart rate of 112 bpm (beats per minute). A transesophageal echocardiogram showed normal global systolic function, mild to moderately dilated atria, moderate mitral and tricuspid regurgitation, and absence of intracardiac thrombus. Prior to cardioversion, she denied chest pain, shortness of breath, orthopnea, or paroxysmal nocturnal dyspnea (PND). Physical exam revealed a normal neck without jugular venous distention (JVD) or carotid bruit, no evidence of rub, no S3-S4, no murmur, and no signs of edema. Cardioversion was performed using 200 J of biphasic mode and with 1 attempt, her AF was converted to sinus bradycardia with a heart rate of 55 bpm. She remained hemodynamically stable and tolerated the procedure well. The following day, the patient was feeling well despite sinus bradycardia and was asymptomatic on ambulation. She was discharged home on amiodarone 400 mg BID and dabigatran 150 mg BID.

Four days post cardioversion, she presented to the emergency room at an outside facility after waking up hearing “wheezes in her chest.” She also experienced substernal chest pressure and shortness of breath while walking. She reported mild weight gain and bilateral lower extremity edema. She denied having orthopnea, PND, nausea, vomiting, diaphoresis, lightheadedness, or radiation of pain to the neck, scapula, or jaw. The patient also denied any increases in stress or emotional state prior to presentation. Physical exam showed normal S1 and S2 heart sounds, 2/6 holosystolic murmur in the mitral position, JVD, and diminished breath sounds bilaterally (left base greater than the right base). She had 1+ bilateral lower extremity pitting edema.

**Figure 1:**
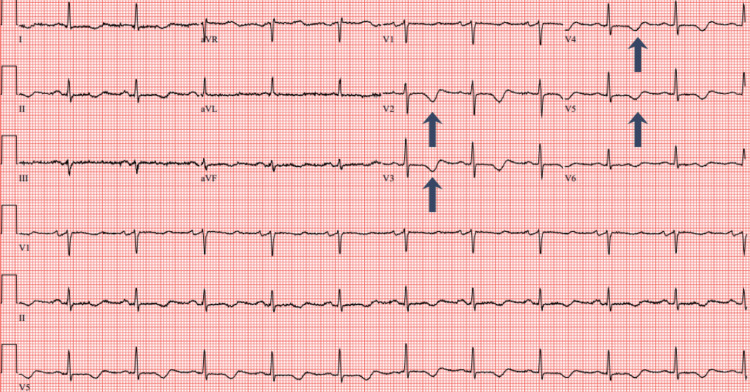
Electrocardiogram showing normal sinus rhythm with T wave inversions in the inferior and anterolateral leads.

**Figure 2:**
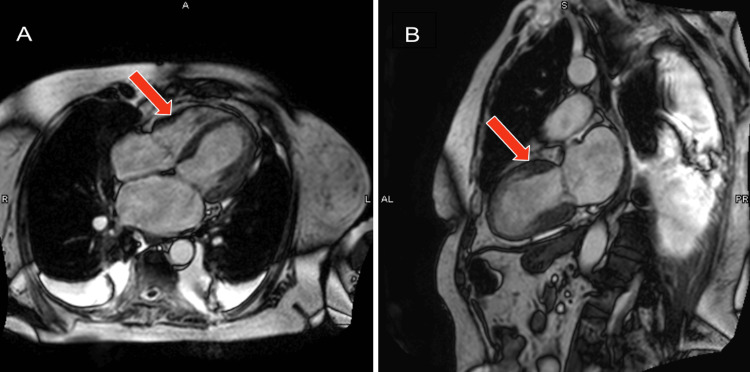
(A and B) SSFP MR images in the four-chamber and two-chamber views during peak systole showing reduced contraction of the apical segments. SSFP: Steady-state free precession; MR: magnetic resonance

**Figure 3:**
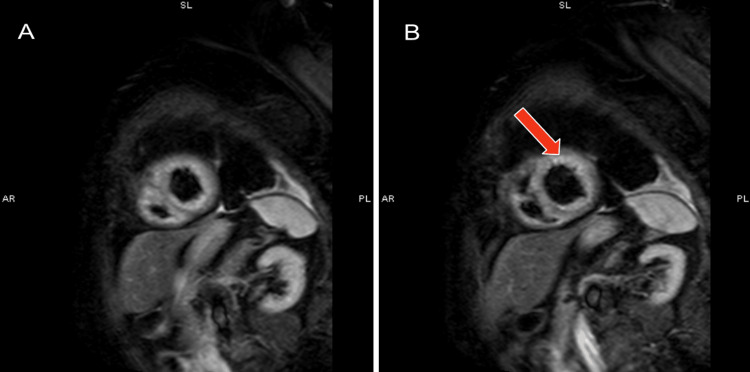
(A and B) T2-weighted MR images of the apical segments of the left ventricle showing evidence of inflammation demonstrated by bright white portions of the myocardium (abnormal) compared to the dark gray portions of the myocardium (normal).

**Figure 4:**
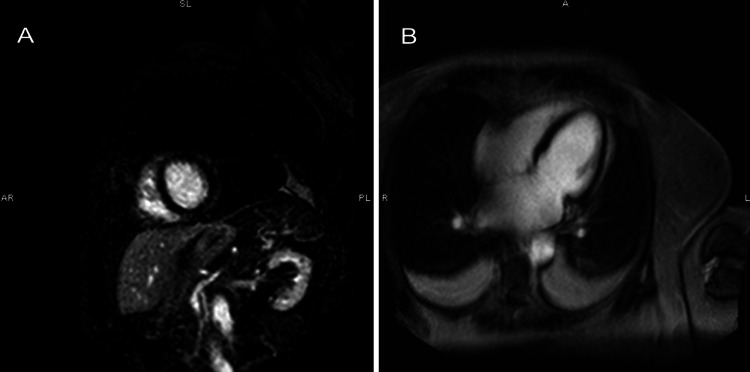
(A and B) LGE imaging MR images showing the absence of significant enhancement in the myocardium including the apical segment where evidence of inflammation was noted. LGE: Late gadolinium enhancement; MR: magnetic resonance

An electrocardiogram (ECG) showed sinus rhythm at 65 bpm, inferior T wave inversions, and anterolateral T wave inversions (Figure 1). Echocardiogram demonstrated a significant decrease in left ventricular ejection fraction (20-25%) with hypokinesis of the basal mid anteroseptal, basal mid inferoseptal, mid apical anterior, mid apical inferior, mid anterolateral, apical septal, apical lateral walls, and the apex. A left pleural effusion was also present. Echocardiogram findings were consistent with LAD infarct or possible atypical TCM. BNP was elevated at 1204 pg/ml and troponin peaked at 0.227 ng/ml. D-dimer was 837 and chest CT (computed tomogram) ruled out acute pulmonary embolism. Coronary angiography showed no signs of obstructive disease. She started on IV furosemide 40mg twice daily, was switched from carvedilol to metoprolol succinate 25 mg daily due to low blood pressure, and continued on amiodarone 200 mg twice daily. Two days after readmission, the patient's shortness of breath resolved and she denied chest pain, lower extremity edema, or orthopnea. A cardiac MRI was performed and it showed an improved left ventricular ejection fraction at 35% (Figure 2). T2 imaging showed evidence of myocardial edema in the apical to mid myocardial segments (Figure 3). T1 mapping showed normal extracellular volume. Late gadolinium enhancement imaging showed faint mid-myocardial enhancement in the basal septal walls (Figure 4). The above findings were consistent with stress-induced TCM. A repeat echocardiogram six days after discharge showed that the left ventricular ejection fraction improved to 45-50%.

## Discussion

TCM, sometimes referred to as “broken heart syndrome” is a temporary heart condition that is characterized by enlargement and reduced function of the left ventricle [1]. Due to the left ventricle’s function as the heart’s main pump, TCM causes the heart to not pump blood as efficiently as it should. Over 90% of reported cases of TCM have occurred in women aged 58-75 years [3]. The exact pathophysiology of TCM is unclear. However, the most widely accepted theories include catecholamine-induced cardiotoxicity and microvascular dysfunction [4]. Diagnosis of TCM is difficult due to the disease's close mirroring of the symptoms of a myocardial infarction (MI). To diagnose TCM, the modified Mayo Clinic criteria can be used and four criteria have to be met: 1) absence of obstructive coronary artery disease (often evaluated by coronary angiography), 2) presence of transient hypokinesis, dyskinesis, or akinesis of the left ventricular mid segments with or without apical involvement, 3) new ECG changes (either ST-segment elevation and/or T-wave inversion) or elevation in serum cardiac troponins, and 4) absence of pheochromocytoma or myocarditis [3]. While both TCM and MI can have ST segment elevations on ECG and elevation in cardiac troponins, the one distinguishing feature of TCM is the transient dysfunction of the left ventricle. The most common abnormality in the left ventricle is the ballooning of the apex of the left ventricle [4]. Additionally, to improve the specificity of TCM diagnosis, the InterkTAK Diagnostic Criteria has been introduced [6]. It includes the following criteria: female sex (25 points), emotional (24 points) or physical trigger (13 points), absence of ST-segment depression (12 points), psychiatric disorders (11 points), neurologic disorders (9 points), and QTc prolongation (6 points) [6]. A score of 50 or greater is highly specific for TCM [6].

Cardiac procedure-induced TCM has been reported in the literature including stress tests and electrical cardioversion [7]. Direct current electrical cardioversion is a procedure used to treat irregular heart rhythms like AF. This procedure is relatively common and involves the use of shocks delivered via electrodes attached to a patient’s chest. This procedure is generally considered to be very safe. The incidence of cardioversion-associated TCM in patients with AF is fairly low. Among 154,919 patients admitted with AF who underwent electrical cardioversion in the National Readmission Database in 2018, 0.027% were readmitted with TCM [5]. Acute heart failure due to apical type TCM was the most common presentation, occurring within 24-48 hours after cardioversion [5]. Generally, the majority of patients were noted to recover within two weeks with supportive care [5].

Only six other cases of cardioversion-mediated TCM have been reported in the literature [8-13]. Four out of six cases involved female patients with a mean age of 77 years [13]. The underlying rhythm prior to cardioversion was AF in the majority of patients (83% of patients) and the time to presentation to ED post-cardioversion ranged from immediately following the procedure to 36 hours [13]. While our patient demonstrated several typical features of TCM such as female gender, postmenopausal age group, and presence of multiple regional wall motion abnormalities including involvement of the apex, our case is the first report of TCM occurring beyond the 48-hour window post-cardioversion. Additionally, unlike most of the previously reported cases of TCM post-cardioversion, our patient did not require any advanced supportive therapy, such as pressors or mechanical ventilation [8-12].

## Conclusions

TCM is a rare complication of electrical cardioversion performed for the treatment of arrhythmias like AF. It often occurs in female patients who are postmenopausal and presents with symptoms of acute heart failure. Though it typically presents within 48 hours post cardioversion, it is important to consider this diagnosis even beyond the two-day window for prompt treatment and prevention of serious sequelae.
